# Linkage to Care for Suburban Heroin Users with Hepatitis C Virus Infection, New Jersey, USA

**DOI:** 10.3201/eid2205.151980

**Published:** 2016-05

**Authors:** Eda Akyar, Kathleen H. Seneca, Serra Akyar, Neal Schofield, Mark P. Schwartz, Ronald G. Nahass

**Affiliations:** ID Care, Hillsborough, New Jersey, USA (E. Akyar, K.H. Seneca, S. Akyar, R.G. Nahass);; University Medical Center at Princeton, Princeton, New Jersey, USA (K.H. Seneca, N. Schofield, M.P. Schwartz, R.G. Nahass);; Rutgers University, New Brunswick, New Jersey, USA (R.G. Nahass)

**Keywords:** Hepatitis C, linkage to care, suburban heroin users, genotype, New Jersey, drug use, viruses, United States

## Abstract

We identified a 41.4% prevalence of hepatitis C virus, absence of HIV, and unexpectedly high frequency of hepatitis C virus genotype 3 among suburban New Jersey heroin users 17–35 years of age during 2014–2015. Despite 2 clinicians prepared to engage these users, few were successfully linked to care and treated.

Hepatitis C virus (HCV) infection is a major public health issue. Although persons born during 1946–1964 represent most of the population with chronic HCV infection, young persons (17–35 years of age) who inject drugs (PWID) now make up the second wave of HCV infection. Up to 90% of all new HCV infections worldwide are attributed to injection drug use; at least 75% of new HCV infections in the United States result from injection drug use (*1*,*2*). In PWID, the prevalence of HCV is 60%–80% (*3*). Often, early age prescription opioid abuse leads to injection drug and heroin use (*2*). Escalating injection drug and heroin use has been associated with increasing HCV infection among young persons (*2*). An estimated 45% of young PWID in the United States are infected with HCV; the annual incidence is 8%–25% (*2*,*4*,*5*).

During 2006–2012 the incidence of acute HCV infections increased significantly among young people in nonurban areas of the United States (*2*). Among those persons, a 13% annual increase of acute HCV infection was reported in nonurban counties, a 170% increase over the course of 6 years (*2*). Young suburban heroin users have been described as the second wave of HCV infection in several US states, including Massachusetts and New York (*5*). New Jersey was not part of those initial reports. Our objective was to characterize HCV infection among young suburban heroin users in New Jersey and to evaluate linkage to care among this population.

## The Study

During October 1, 2014–June 9, 2015, Princeton House, a psychiatric facility in suburban New Jersey with an active opioid detoxification program, instituted a new HCV screening program. As part of the standard of care, patients admitted for heroin detoxification were tested for HIV, HCV, and hepatitis B virus infections. The average length of stay for opioid detoxification at Princeton House is 6 days. All patients positive for HCV antibody were clinically evaluated. HCV viral load and reflex genotypes (GTs) were obtained. Follow-up visits at Princeton House before discharge were performed by one of the authors (K.H.S. or R.G.N.) to counsel patients on results and the disease and to link patients to care by providing directions and appointments to HCV caregivers near patients’ homes.

A total of 861 unique patients from 10 of 21 New Jersey counties were tested for HCV antibody; 374 (43.4%) were positive. Most (573 [66.6%]) patients were 17–35 years of age. Of those, 237 (41.4%) were HCV antibody positive. From this population, 187 patients were further evaluated; 50 patients refused evaluation or were discharged before evaluation. Women constituted 52.4%. Races and ethnicities were 173 non-Hispanic white, 2 non-Hispanic black, 4 Hispanic, and 8 other.

HCV viral load was obtained for 172 (92.0%) of the 187 patients; 15 patients were missed or not properly collected. For 32 (18.6%) patients, viral load was undetectable. HCV GTs were obtained from 102 patients: 64 (62.7%) were GT1a, 3 (2.9%) were GT1b, 8 (7.8%) were GT1 undefined, 1 (1.0%) was GT2, and 26 (25.5%) were GT3. Eight patients were identified with acute HCV. All patients who were HCV antibody positive were HIV antibody negative. 

Of the 187 patients, 16 (8.6%) had outpatient follow-up appointments, and 3 (1.6%) started oral, direct-acting antiviral treatment. Two of the 3 patients failed to adhere to treatment regimen. One of the 16 patients spontaneously cleared infection before drug-treatment initiation. Two other patients returned for treatment but were denied prescriptions by insurance; all others failed to return for continued care.

## Conclusions

Our study indicates that HCV was highly prevalent in young suburban heroin users attending an acute detoxification program that serves a wide geographic area, suggesting that New Jersey is participating in the second wave of HCV infection. Our study highlights the challenges of linking young PWID in suburban areas to care despite the effort of 2 clinicians with extensive HCV experience to engage patients in the care cascade. That most patients were women (52.4%) and non-Hispanic white (92.5%) probably reflects the demographic of persons seeking detoxification from heroin and coincides with demographics of other reports of young nonurban PWID in the United Sates (*2*).

The 25.5% (95% CI 17%–34%) prevalence of GT3 among this population of young suburban heroin users is more than twice the national average of 12% (*6*). This pattern of distribution suggests a closed network of injection drug users engaging in risky behavior that leads to HCV transmission.

Although HCV screening was easily attainable in Princeton House, linking patients to care was a challenge (Figure). Even with encouragement, only 16 (8.6%) patients returned for in-office follow-up visits, and 2 started treatment. Patient follow-up after patients left Princeton House was a logistical challenge because of patient relocation and availability of transportation and communication. The program of acute detoxification at Princeton House simply withdrew patients from heroin under direct observation. Long-term patient management required additional treatment for patient addiction upon discharge, which was difficult to achieve. We were also limited by the absence of a care coordinator to assist with the linkage to care effort. Finally, some patients who came for outpatient treatment were denied treatment by payers because of the requirement of a clean drug test before treatment initiation or because patients did not have advanced liver disease, defined as Stage 3 or 4. All create additional complexity, which delays treatment. Because we did not formally assess patients’ psychosocial circumstances, our considerations for the reasons for failure to link to care are speculative and are currently being studied. We believe that difficulty in linkage to care represents an area where greater support will be critical, such as through the use of case management, as was done with HIV care under the Ryan White program (*7*).

**Figure:**
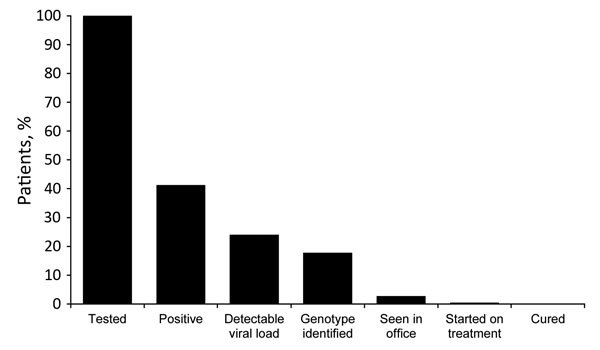
Cascade of care for suburban heroin users 17–35 years of age, New Jersey, October 1, 2014–June 9, 2015.

Also noteworthy is the absence of HIV in a population with a high percentage of injection drug use in a state with a high prevalence of HIV. Indiana’s recent outbreak of HIV among young heroin users increases concern for the establishment of HIV in this network (*8*). To achieve success similar to that of HIV treatment in PWID, a coordinated program that includes committed case management services to help PWID navigate the complexity of accessing and maintaining treatment is likely to be needed. Further study to explore this and the ability to successfully treat this population is crucial to address the national HCV infection epidemic.

Finally, a misconception exists that PWID are poor candidates for treatment because of ongoing drug use, possible reinfection, and possible concomitant psychiatric or medical disorders (*9*). However, recent reports suggest that such patients can be successfully treated with newer therapies (*10*). Success in reducing HIV transmission among PWID during the past decade provides evidence that infected drug users can achieve adherence levels similar to persons who do not use drugs (*9*,*11*). The potential for treatment as prevention was discussed by Hellard et al., who calculated the minimum number of patients within a network needed to be treated to reduce or eliminate transmission (*10*). Given the availability of easy-to-use therapy that is curative, linkage to care and treatment of HCV-infected PWID may be an important public health effort to prevent the continued spread of HCV. Further study is needed to identify predictors for successful linkage to care.
